# Statistical Inference Methods for Sparse Biological Time Series Data

**DOI:** 10.1186/1752-0509-5-57

**Published:** 2011-04-25

**Authors:** Juliet Ndukum, Luís L Fonseca, Helena Santos, Eberhard O Voit, Susmita Datta

**Affiliations:** 1 Department of Bioinformatics and Biostatistics, School of Public Health and Information Sciences, University of Louisville, Louisville, KY 40202, USA; 2 Instituto de Tecnologia Química e Biológica, Universidade Nova de Lisboa, Rua da Quinta Grande 6, 2780-156 Oeiras, Portugal; 3 Integrative BioSystems Institute and Wallace H. Coulter Department of Biomedical Engineering, Georgia Institute of Technology and Emory University, 313 Ferst Drive, Atlanta, GA 30332, USA

## Abstract

**Background:**

Comparing metabolic profiles under different biological perturbations has become a powerful approach to investigating the functioning of cells. The profiles can be taken as single snapshots of a system, but more information is gained if they are measured longitudinally over time. The results are short time series consisting of relatively sparse data that cannot be analyzed effectively with standard time series techniques, such as autocorrelation and frequency domain methods. In this work, we study longitudinal time series profiles of glucose consumption in the yeast *Saccharomyces cerevisiae *under different temperatures and preconditioning regimens, which we obtained with methods of *in vivo *nuclear magnetic resonance (NMR) spectroscopy. For the statistical analysis we first fit several nonlinear mixed effect regression models to the longitudinal profiles and then used an ANOVA likelihood ratio method in order to test for significant differences between the profiles.

**Results:**

The proposed methods are capable of distinguishing metabolic time trends resulting from different treatments and associate significance levels to these differences. Among several nonlinear mixed-effects regression models tested, a three-parameter logistic function represents the data with highest accuracy. ANOVA and likelihood ratio tests suggest that there are significant differences between the glucose consumption rate profiles for cells that had been--or had not been--preconditioned by heat during growth. Furthermore, pair-wise *t*-tests reveal significant differences in the longitudinal profiles for glucose consumption rates between optimal conditions and heat stress, optimal and recovery conditions, and heat stress and recovery conditions (*p*-values <0.0001).

**Conclusion:**

We have developed a nonlinear mixed effects model that is appropriate for the analysis of sparse metabolic and physiological time profiles. The model permits sound statistical inference procedures, based on ANOVA likelihood ratio tests, for testing the significance of differences between short time course data under different biological perturbations.

## Background

Innovations in molecular biology, miniaturization, and robotics have led to genetic, proteomic and metabolomic data in quantities never seen before. Microarrays exhibit the expression state of thousands of genes in a single experiment, 2-D gels and other proteomic methods identify subtle changes in the protein profile of an organism under altered conditions, while techniques of mass spectrometry are capable of quantifying hundreds of metabolites in one spectrogram. Most of these new tools have been utilized by biologists to obtain snapshots of the state of a cell population, and these snapshots have been compared to controls in order to gain insights into the responses of cells to various stimuli or perturbations. Thus, a typical experiment might compare the gene expression in a cancer cell to the corresponding expression profile in normal, healthy cells.

Comparisons between molecular profiles of perturbed cells and controls have vastly extended our understanding of the healthy and pathological functioning of cells. Their drawback is the singular nature of measurements at only one time point, which limits insights into the scope of a response and leaves doubt whether the best time point had been selected for data collection. Responding to this disadvantage of snapshot comparisons, recent technical advances in molecular biology have rendered it possible, as well as practically and economically feasible, to execute high-throughput measurements in time series. For instance, many data are now available characterizing the genomic responses of microorganisms to a variety of stimuli over a period of time [1]. Similarly, non-invasive **nuclear magnetic resonance **(NMR) measurements can characterize the time development of metabolites in intact cells over a span of hours, with data taken every minute or in some cases even every 10 to 20 seconds [2]. These time series data contain enormous amounts of information, because they are systemic as well as dynamic and capture the cellular response in a more comprehensive fashion than measurements at single time points can achieve. Furthermore, time series data constrain each other in some sense, for instance by facilitating the identification and interpretation of outliers and measurement errors.

Biological time series data contain more information than snapshot data but they cannot necessarily be evaluated with the typical methods of time series analysis, such as autocorrelation and frequency domain techniques, because these were often developed for much longer and denser time series, as they are found, for instance, in weather recordings. In comparison to such data, presently available biological data are very sparse. Indeed, many time series consist of maybe a dozen quantities, measured at a handful of time points, and replicates are usually scarce. This paucity of data, accompanied by minimal redundancy, immediately creates statistical challenges that have not been addressed in a systematic fashion. For instance, in the analysis of microarray time series [3], genes are often merely clustered visually into time trends, such as monotone increase/decrease, S-shaped increase/decrease, or initial increase/decrease followed by a gradual return to the initial state. For metabolic time series, even fewer methods are available, and it is not often possible to state with a sufficient level of confidence and objectivity whether two time series are significantly different.

We encountered this issue in the analysis of *in vivo *NMR time series measurements in the baker's yeast *Saccharomyces cerevisiae *[4] and the lactic acid bacterium *Lactococcus lactis *[5]. Investigating these systems under many different conditions, we found it difficult to decide how much of the differences between two time courses were due to normal variation between cell cultures and experimental settings and how much was pointing toward true differences in the cells' response dynamics. We therefore decided to develop sound statistical methods for assessing the significance of differences between biological time series.

Specifically, we analyzed the following situation. We grew yeast cells under optimal conditions to a particular population density and measured the uptake of their favorite substrate, glucose, over time. We compared these time-dependent uptake profiles with those of the same cells exposed to persistent heat, which in yeast evokes a systemic heat stress response. We then returned the cells to optimal temperature and measured the uptake dynamics under recovery conditions. The simple question we asked was whether the uptake characteristics are significantly different among the three situations of ambient optimal temperature, heat, and recovery. In addition, we were interested in answering the question of whether preconditioning the cells with heat during growth would affect their responses to heat later in life.

While the biological implications are of relevance in themselves, the more important focus here is on more or less generic analytical tools for scarce time series in biology. Thus, we propose in the following the adaptation of statistical techniques to the significance analysis of differences between relatively sparse biological time series data.

## Methods

### Biological Methods

#### Strain and growth conditions

*Saccharomyces cerevisiae *strain JK93dα was kindly supplied by Dr. Ashley Cowart, Medical University of South Carolina. Cells were grown up to an OD_600 _(cell density) of approximately 2 in G0 minimal medium [6] supplemented with 0.2% yeast extract, in a 5-liter fermentor (100 RPM, pH 6.5 under continuous flushing with air, pO_2 _> 80%). We compared two sets of preconditions. Under control conditions, the cells were grown at an optimal temperature of 30°C throughout, whereas cultures preconditioned by heat stress were prepared as follows: cells were initially grown under control conditions (30°C) up to an OD_600 _of approximately 1.3; at this point the temperature was increased to 39°C (heat stress) and the cells were allowed to grow for further 40 min to a final OD_600 _of about 2. The actual experiments followed afterwards.

#### Online measurements of glucose uptake by carbon-13 NMR (^13^C-NMR)

Cells were harvested, centrifuged (10 min, 8,600 × *g*, 4°C), washed (5 mM potassium phosphate buffer (KP_i_), pH 6.5), and re-suspended in 50 mM KP_i _buffer containing 6% (v/v) ^2^H_2_O and antifoam agent. The cell suspension (typically 60 mg dry weight/mL) was transferred to an 80-mL fermentor, maintained at 30°C, and connected to a 10-mm NMR tube by a circulating system [7]. The culture was pumped through the NMR tube at a rate of 30 mL/min and the pH was maintained at 6.5 by automatic addition of NaOH or HCl. At time zero, [1-^13^C] glucose (65 mM final concentration) was added and the glucose concentration monitored by ^13^C-NMR until substrate depletion, with a time resolution of 30 seconds. The glucose was typically consumed within 10 minutes, and the cells began to starve for about 20 minutes. Afterwards, the temperature was raised to 39°C, and a second pulse of glucose was added. Once glucose was exhausted again, the temperature was set back to 30°C and a third pulse of glucose supplied. The ^13^C-NMR time series were acquired on a Bruker DRX500 spectrometer using a quadruple-nucleus probe head as previously described [6].

#### Experimental Data

Six glucose uptake experiments were performed. They were essentially the same, but differed slightly in the amount of biomass at harvesting and, more importantly, in the temperature of the medium, which was initially optimal (30°C), then elevated to heat stress (39°C), and again reverted back to optimal (30°C) during recovery after heat stress. In the following, the experiments will be denoted as NG1 (normal growth; 83.85 mg/ml), NG2 (normal growth; 57.05 mg/ml), NG3 (normal growth; 60.3 mg/ml), NG4 (normal growth; 61.55 mg/ml), PC1 (preconditioned; 73.45 mg/ml), and PC2 (preconditioned; 42.8 mg/ml), where the values in parentheses correspond to the biomass concentration used in each experiment.

**Figure 1:**
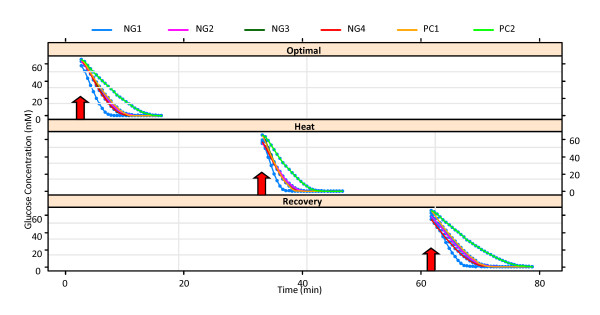
**Glucose uptake profiles for the six experiments under optimal temperature, heat stress, and recovery temperature**.

**Figure 2:**
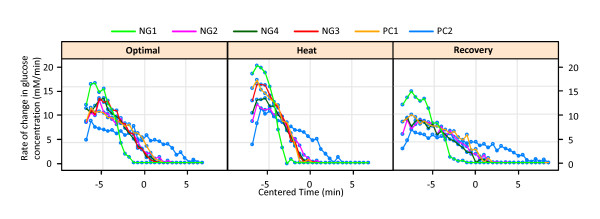
**Rate of change of glucose concentration (mM/min) with respect to centered time (in minutes); under the three different temperatures 30°C (optimal), 39°C (heat stress) and 30°C (recovery) for the six different experiments**.

The cultures for experiments NG2, NG3, and NG4 were grown to the target optical density (OD) of 2.0 and hence the respective biomasses are similar. Cells for NG1 were grown under the same conditions but harvested later (OD = 2.8), thereby starting the actual experiments with a larger population size. As a consequence, the substrate was used up faster in this case. In order to check whether there are differences in future metabolic profiles of the cells which are grown under stressful conditions some of the cells (experiments PC1 and PC2) were heat stressed during the final 40 minutes of growth prior to the *in vivo *NMR experiments (see also [4]). In all experiments, the glucose concentrations were recorded at equal time intervals of 30 seconds, starting roughly at times 3, 33 and 63 minutes for optimal, heat stress and recovery conditions, respectively. Figure 1 shows the original glucose consumption curves, stating when glucose was added (arrows), and Figure 2 the corresponding rates of change in glucose concentration for each experiment for different time points. As explained below, the time profiles were centered for the statistical analysis.

### Statistical Methods

#### General objective

The main objective of this study is to develop statistical tools that allow a biologist to decide whether differences in similar time courses (here, the change of glucose concentrations with respect to time) are statistically significant (here, with respect to different temperatures of the medium and preconditioning) or simply due to experimental and biological noise or intrinsic variability. This objective is generically of great interest in systems biology, because it directly affects the construction of mechanistic models underlying the investigated time courses [4]. In particular, the statistical outcome of the analysis forms the basis for the inclusion or exclusion of variables in such models, and statistical significance will directly lead to new hypotheses as to what biological factors or mechanisms might be causing the differences.

In the illustrative example here, the secondary specific task is to determine whether environmental (heat) preconditioning during population growth affects the dynamics of glucose uptake later in the life of the yeast cultures. As expected, glucose consumption increases with the amount of biomass. However, an unanswered question is whether the uptake profile is significantly different under altered environmental conditions. Thus, we intend to analyze the following questions: First, if one adjusts for the differences in initial biomass, are the glucose uptake profiles essentially the same or do they significantly differ under optimal, heat stress and recovery conditions? And secondly, do the uptake profiles differ between cells that were either grown under optimal conditions (30°C) or preconditioned by heat stress (39°C) during growth? These questions are not trivial to answer, because standard methods of statistics are not usually designed to test hypotheses regarding entire time profiles for data available at only few time points.

#### Formulation of specific hypotheses

The first specific hypothesis of interest is that the dynamic profile of glucose consumption depends on the current ambient temperature. The second hypothesis is that glucose uptake profiles for cells grown under optimal conditions differ from the cells grown under heat stress conditions (preconditioning). In order to test these hypotheses, we need to select a stochastic model that fits the dynamics of the rate of change of glucose concentration with respect to time. Effects caused by moderate differences in initial biomass are accounted for by formulating this factor as a covariate in the stochastic models.

To facilitate the selection of a suitable stochastic model for the glucose uptake dynamics, we first plot the rate of change of glucose concentration (mM/min) with respect to *centered time*, *i.e*., the difference between the actual time recorded and the mean time of the experiment (in minutes), under the three different temperatures 30°C, 39°C, 30°C for all experiments (Figure 2). These plots indeed seem to suggest that initial biomass (see dataset D1) as well as both the ambient temperature and the preconditioning with heat during growth might have an effect on the glucose uptake dynamics. Specifically, it appears that heat stress is associated with a higher rate of change in glucose concentration.

Although there are considerable variations in the uptake curves among experiment types, essentially all the plots follow a reverse S-shape if the first one or two data points are ignored. These first points correspond to a delay of uptake initiation that is caused by the experimental set-up and the fact that the cell population has to adjust to the new conditions. In the following, the early data points will be included in the analysis, but the initial rise in the rate of glucose uptake will not be considered as structurally important.

#### Model selection

It is evident from the plots of the data that a nonlinear model is required to capture the dynamics of the time courses. Accommodating this requirement, as well as statistical feasibility, we chose nonlinear mixed-effects (NLME) models for our analysis, which are best known for analyses of repeated measures and, in particular, longitudinal data. In NLME analysis, some or all of the fixed and random effects occur nonlinearly in the model function. NLME models can be regarded as extensions of linear mixed-effects models in which the conditional expectation of the response, given the fixed and random effects, is allowed to be a nonlinear function of the coefficients. At the same time, NLME models can also be viewed as extensions of nonlinear regression models for independent data, in which random effects are incorporated in the coefficients to allow them to vary by experiment type (hereafter referred to as *type*), thus inducing correlation within the types. The latter aspect is particularly pertinent for our modeling task.

The NLME model for repeated measures proposed by [8] can be thought of as a hierarchical model, where the *j^th ^*observation in the *i^th ^*type and corresponding to *k^th ^*level of a covariate is modeled as(1.1)

Here *M *is the number of experimental types, *n_i _*is the number of observations on the *i^th ^*type, *l *describes the levels of the primary covariate, *f *is a general, real-valued, differentiable function with a type-specific parameter vector φ*_ij _*and a covariate vector *ν_ijk_*, and ε*_ijk _*is a normally distributed within-type error term. The function *f *is nonlinear in at least one component of the type-specific parameter vector φ*_ij_*, which is modeled as(1.2)

In this formulation, β is a *p*-dimensional vector of fixed effects and *b_i _*is a *q*-dimensional random effects vector associated with the *i^th ^*type with variance-covariance matrix ψ. The matrices *A_ij _*and *B_ij _*are of appropriate dimensions and depend on the type and possibly on the values of some covariates at the *j^th ^*observation.

The model (1.2) was first proposed in [9]. It assumes that observations corresponding to different experimental types are independent and that the within-type errors ε*_ijk _*are independently distributed as *N*(0,σ^2^) and independent of the *b_i_*. However, if necessary, this assumption of independence and homoscedasticity for the within-type errors can be relaxed.

In accordance with the general shape of the time course data, we postulate three possible nonlinear models for *f*, namely a simple exponential decay curve, a three-parameter logistic model, and a four-parameter logistic model.

#### Notation

As in Eq. (1.1), let *i *denote the index for the experiment type, and *j *the index for the observation number within each experiment type. Thus *i *= 1,2,3,4,5,6 represents the six experiment types and *j *= 1,2,...,*n_i_*, where, *n_i _*is the number of observations in the *i^th ^*experiment type. Furthermore, let *y_ij _*denote the rate of change of glucose concentration for experiment type *i *of the *j^th ^*measurement and *t_ij _*the centered time recorded for the *i^th ^*experiment type and the *j^th ^*measurement. In the mathematical representation below, as well as in the model building and selection process, we initially ignore the effects due to temperature and preconditioning during cell growth. However, the effect of these covariates, namely temperature, preconditioning and the temperature-preconditioning interaction, is incorporated in the best model and presented later (see Eq. 3.5).

##### Model 1: Exponential Decay Curve

The exponential model has plenty of theoretical and empirical support. In particular, if the dynamics of glucose (*y*) followed a simple transport process (from the medium into the cells) or a first-order kinetic process, it would be described as a linear differential equation of the type , whose solution and rate of change are exponential functions. Although, the exponential decay model is common for the first-order kinetic equations, it can fail outside the range of the data. Hence we chose instead a more flexible model with more theoretical and empirical support, namely a three-parameter exponential decay curve that does not necessarily have zero as its asymptote. Thus, in this first case we express the rate of change of glucose concentration *y_ij _*of experiment type *i *at time *t*_*ij *_for *i *= 1,2,3,4,5,6 *j *= 1,2,..., *n*_*i*_(2.1)

The function contains three parameters, of which, λ_1 _is the right asymptote, λ_2 _is the total amount of glucose ultimately taken up, and λ_3 _is the time needed to take up half of the maximum amount [10]. ε*_ij _*is a normally distributed (*N*(0,σ^2^)) error term, which is assumed to be identically and independently distributed (i.i.d.). λ_1 _and λ_2 _are linear parameters while λ_3 _is a nonlinear parameter. Additional file 1 Table S1 in the Appendix, shows that the three-parameter model fits the data significantly better than the simple two-parameter model.

##### Model 2: Three-parameter Logistic Model

The three-parameter logistic model expresses the rate of change in glucose concentration *y_ij _*of experiment type *i *at time *t_ij _*as(3.1)

Like the previous one, this model is governed by three parameters. λ_1 _is the maximum rate of change for glucose concentration λ_2 _is the time at which the maximum rate of change for glucose concentration attains half of its maximum value, and λ_3 _is the reciprocal of the decay rate. (As generic support for the appropriateness of this model, see [9] and [10]). As before, ε*_ij _*is an i.i.d. *N*(0,σ^2^) error term. λ_1 _is a linear parameter, while λ_2 _and λ_3 _are nonlinear parameters.

The format in Eq. (3.1) is not often seen in biochemistry, where nonlinear models are preferred if they can be interpreted mechanistically and if their parameters have a natural physical interpretation. For instance, a typical description of the glucose uptake process might be in the form of a kinetic Michaelis-Menten process of the type(3.2a)

**Figure 3:**
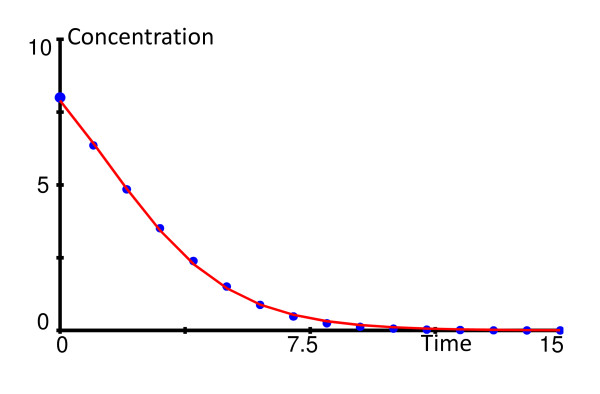
The three-parameter logistic model (3.1; solid line) provides an excellent approximation for the solution of the Michaelis-Menten model (symbols) in (3.2*). See text for parameter values.

This differential equation can be solved analytically, but the explicit solution of *y *as a function of time is rather convoluted. However, the simpler three-parameter logistic model (3.1) offers a very good approximation. For instance, if the parameters of the Michaelis-Menten function (3.2*) are arbitrarily chosen as *V*_max _= 2.4 *K_M _*= 3.3 and *y*(0) = 8, the logistic model (3.1) with λ_1 _= 11.6, λ_2 _= 1.4 and λ_3 _= -1.85 results in a graph that is essentially indistinguishable from the solution of the differential equation in (3.2*) (Figure 3). Thus, within minimal experimental noise, the simple three-parameter logistic function models a Michaelis-Menten-type uptake function with sufficient accuracy.

##### Model 3: Four-parameter Logistic Model

The four-parameter logistic model is given by the mathematical formula(4.1)

Here, λ_1 _is the horizontal right asymptote, λ_2 _is the horizontal left asymptote (baseline) and λ_3 _is the time that corresponds to the rate of glucose consumption halfway between the asymptotes ([9] and [10]). λ_4 _is the reciprocal of the decay rate and ε*_ij _*is again an i.i.d. *N*(0,σ^2^) error term. In Eq. (4.1), λ_1 _and λ_2 _are linear parameters, while λ_3 _and λ_4 _are nonlinear parameters. To resolve the issue of lack of identifiability, we require that λ_4 _must be strictly positive. Clearly, (3.1) is a special case of (4.1) with λ_1 _= 0.

The parameters of the models (2.1), (3.1), and (4.1) are obtained with nonlinear least squares (*nls*) regression. To fit a separate nonlinear model to each dataset and thus allowing the individual effects (experiment types including temperatures) to be incorporated in the parameter estimates, we express the models (2.1), (3.1) and (4.1) as(2.2)(3.2)(4.2)

where, as before, the ε*_ij_*'s are i.i.d. *N*(0,σ^2^) errors. The functions were subsequently used in our NLME model with a mixture of fixed and random effects. In the statistical software package R [11], models (2.2), (3.2), and (4.2) are considered *nlsList *models, since their parameters are estimated with nonlinear least squares methods.

To account for fixed and random effects in a nonlinear mixed-effects model, it is useful to reformulate the models (2.2), (3.2) and (4.2) respectively as(2.3)(3.3)(4.3)

where the  quantities denote the means of the parameters for each type. In *nlsList *model(s), the deviations of the coefficients from their means are treated as parameters that need to be estimated. Mixed-effects models, on the other hand, represent these deviations from the mean value as random effects, treating each of the types as a sample from a population. The nonlinear mixed-effects versions of (2.2), (3.2) and (4.2) are therefore(2.4)(3.4)(4.4)

The fixed effects β_1_,β_2_,β_3 _(and β_4_) represent the mean values of the parameters for each type, λ*_i _*is the population of experiment types and the random effects, *b*_*i*1_,*b*_*i*2_,*b*_*i*3 _(and *b_i_*_4_), represent the deviations of the λ_i _from their population average. λ*_i_*'s contain both fixed and random effects. The random effects are assumed to be distributed normally with mean 0 and variance-covariance matrix ψ. Random effects corresponding to different types are assumed to be independent. The within-type errors ε*_ij _*are assumed to be independently distributed as *N*(0,σ^2^) for different *i*, *j *and to be independent of the random effects. Models (2.4), (3.4), and (4.4) are considered nonlinear mixed-effects (NLME) models since both fixed and random effects are incorporated in the models. The nonlinear mixed-effects models (2.4), (3.4), and (4.4) offer a compromise between the rigid nonlinear least squares (*nls*) models (2.1), (3.1) and (4.1) and the over-parameterized *nlsList *models (2.2), (3.2) and (4.2). They accommodate variations for each experimental type through the random effects, but tie the different types together through the fixed effects and the variance-covariance matrix ψ. A crucial step in the model-building of mixed-effects models is deciding which of the coefficients (parameters) in the model need random effects to account for their between-type variation and which can be treated as purely fixed effects.

We now fit a nonlinear regression model for the rate of change of glucose concentration with respect to time for each temperature. For this purpose, we require for each temperature the same starting time for glucose uptake. Since we are interested in modeling and drawing statistical inferences for the rate of change in glucose concentration and the collection times are equally spaced for each temperature, we consider the starting times for each temperature to be the ones for which the first glucose concentration data are available. Furthermore, it simplifies the computation, without loss of generality, if we use centered times, by considering the differences between recorded times and the mean time of the experiment type as in Figure 2.

Initially ignoring the effects of temperature, we fit the nonlinear least squares model of the data to determine which of the three model functions ((2.1), (3.1) or (4.1)) best fits the time trends in the data. Then we fit nonlinear least squares curves for each experiment type in order to determine if the models require random effects (*nlsList *models (2.2), (3.2), (4.2)). The main purpose of this preliminary analysis provided by *nlsList *is to suggest a structure for the random effects to be used in a nonlinear mixed-effects regression model. Another important advantage of using an *nlsList *object is that it automatically provides initial estimates for the fixed effects, the random effects, and the random-effects covariance matrix.

Following the model-building process for nonlinear mixed-effects models proposed by [9], we first checked all assumptions regarding the random effects, tested for heteroscedasticity of the within-type error variance and the correlation of the random effects. We then performed a likelihood ratio test, and checked the Akaike Information Criterion (AIC) as well as the Bayesian Information Criterion (BIC) to select the nonlinear regression model that best fits the data. In this case, it turned out to be the three-parameter logistic model. The model parameters were obtained with the method of maximum likelihood estimation, and the parameter estimates are reported by fitting the best nonlinear regression model with fixed effects terms; temperature, preconditioning and the temperature-preconditioning interaction included in the model as covariates. Thus, the mathematical formula for the three-parameter logistic nonlinear model with temperature, preconditioning and temperature-preconditioning interaction term as covariates for all the parameters is given by(3.5)

where, as before,β_1_, β_2 _and β_3 _represent the mean values of the parameters for each type λ*_i _*in the population of experiment types and the random effects, and *b_i_*_1_,*b*_*i*2 _and *b*_*i*3 _represent the deviations of the λ*_i _*from their population average. Furthermore, let *k *denote the index for the covariate temperature used while observing glucose consumption. Thus, *Temp_k _*represents the specific temperature index *k *= 1,2 that corresponds to heat stress or recovery conditions, respectively. Note that the temperature 'optimal' is used as reference category. The index *precond *represents the two preconditioning levels corresponding to either optimal or heat stress conditions during the late growth of the cell populations, before the actual experiments started. γ_11_, γ_21 _and γ_31 _(γ_12_, γ_22 _and γ_32_) represent the coefficients for the difference between the optimal temperature category and heat stress (or recovery). θ_1_, θ_2 _and θ_3 _represent the coefficients for the difference between optimal condition and preconditioning during initial growth. ξ_11_, ξ_21 _and ξ_31 _(ξ_12_, ξ_22 _and ξ_32_) represent the coefficients for the interaction between the heat stress-preconditioning and the temperatures while observing glucose consumption.

### Methods for Testing the Temperature Effect

Analysis of variance (*ANOVA*) was used to test if temperature has an effect on the glucose dynamics. For detecting the overall difference in the dynamics due to differences in temperatures, we considered two models, namely a *full model*, in which parameters of the nonlinear regression model vary with respect to temperature and biomass concentration, and a *reduced model *with constant and temperature independent parameters. The likelihood ratio test was performed for assessing the overall temperature effect, and characterized with a *p*-value.

For pair-wise comparisons, we considered appropriate subsets of the data. Initially, we used ANOVA with the approximate *F*-test to test the joint significance of the fixed effects introduced in the model. The *F*-test for joint significance was performed, and *p*-values were reported for the test as well.

When using the *F*-test, the assumption of normality should be justified. However, it is extremely difficult to check for normality in the case of nonlinear mixed-effects models. In fact, as the random effects are themselves not observable, a direct check of their normality is not possible. Moreover, the complicated way in which the random effects enter a nonlinear mixed model makes it impossible to check the assumption of normality of the errors. An interesting discussion on this specific topic can be found in Hartford and Davidian (see [12]). These authors mention the fact that, because the random effects are unobservable latent model components, no straightforward diagnostic is available to check the validity of the normality assumption. However, huge simulations have shown that estimation of fixed parameters may not be severely compromised (*e.g*., [13]) even if the assumption of normality of the random effects is violated.

### Methods for Testing the Effect of Preconditioning

To investigate the effect of heat preconditioning during growth, we used a similar procedure as before for testing the effect of temperature on glucose uptake dynamics, namely the ANOVA likelihood ratio test. In addition, the approximate *F*-test was used for testing the joint significance of fixed effects terms in the model.

### Simulation Study

To illustrate and justify the overall usefulness of the proposed method of fitting a three-parameter logistic model to the glucose uptake profiles we carried out a robustness analysis with the help of a simulation study. The steps in the simulation study were as follows:

In the first step, we simulated * i *= 1,2,3,4,5,6; *j *= 1,2,...,*n_i_*. We used the estimated value of σ = 0.3103 from the dataset. We further simulated the random effects . We used multiples of the estimates of the variance-covariance matrix of the random effects as obtained from the real data as ψ to generate the simulated data. We also used the parameter estimates of the fixed effects coefficients directly from the data to generate the simulated data. Thus, the simulated data  were generated from the model

A sample size of 1000 was used. The parameters were estimated from each simulated data set. We systematically changed the random effects variances ψ, simulated data sets multiple times, and estimated the parameters of the three-parameter logistic model for every instance. Specifically, parameter estimates of the fixed effects terms, bias and standard error for different choices of ψ's are reported.

## Results

### Results for Model Fitting and Statistical Methods of Significance Analysis

#### Model Fitting

**Figure 4:**
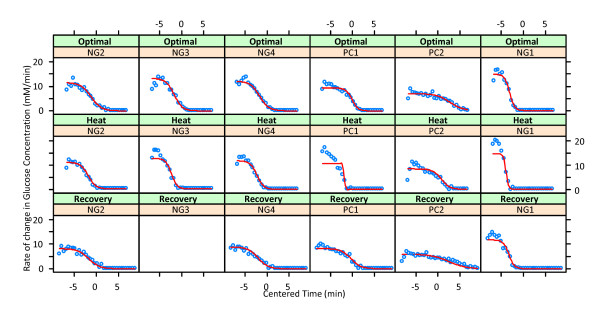
**Plots of observed data (blue circles; adjusted) and time courses (red lines) obtained from a simultaneously fitted three-parameter logistic model in the implementation of a reduced model of Eq. (3.5) with temperature as the only covariate**. No measurements were taken during recovery in experiment NG3. Notice that NG1 (right column), while normally grown, started with a substantially higher biomass than NG2-NG4. PC1-PC2 indicate preconditioned cells.

We employed all NLME models with the three-parameter exponential and the two logistic functions to fit all data simultaneously. Among them, the three-parameter logistic model had the lowest AIC and BIC values of 1911.637 and 1953.981, respectively (see Additional file 1 Table S2, Additional file 1 Table S3, and Additional file 1 Table S4 in the Appendix). All but one of the parameter estimates (intercept not included) of this model were found to be significant at the 5% significance level (Table 1), indicating that the three-parameter logistic model provides overall the best simultaneous fit for all data. Indeed, the logistic mixed-effects nonlinear regression model fits the glucose uptake dynamics through the rate of change of glucose concentration rather well for all given datasets (Figure 4). The (initial) temperature condition 'optimal' is considered as the reference temperature and referred to as the *intercept of the model*.

**Table 1:** Parameter estimates for the three-parameter logistic model with temperature included in the model as covariate.

Parameter	Estimate	t-value	*p*-value
β_1_.(Intercept)	10.4096	16.1326	0.0000

β_1_.Heat	0.6456	1.1436	0.2533

β_1_.Recovery	-2.0912	-4.1018	0.0000

β_2_.(Intercept)	-0.8622	-1.5464	0.1227

β_2_.Heat	-0.9627	-6.9235	0.0000

β_2_.Recovery	-0.7040	-3.2702	0.0011

β_3_.(Intercept)	-1.0846	-6.0598	0.0000

β_3_.Heat	0.5000	6.4934	0.0000

β_3_.Recovery	-0.3239	-2.6566	0.0081

The optimal condition is considered as the reference temperature.

The intercept terms (β_1_.Intercept, β_2_.Intercept, and β_3_.Intercept) represent the mean parameter estimates for the optimal temperature condition for the six experiment types. Specifically, β1.Intercept is the maximum value for the rate of change of glucose concentration for the optimal temperature averaged over the six experiment types. For the optimal temperature, β2.Intercept is the time at which the uptake rate curve attains half of its maximum value averaged over the six experiment types. The estimated β3.Intercept parameter represents the average rate of glucose utilization in the six experiment types for the optimal temperature.

The parameter estimates associated with an increase in temperature from optimal to heat stress for the six experiment types are quantified by β_1_.Heat, β_2_.Heat and β_3_.Heat. The parameter estimate β_1_.Heat, on average for the six experiment types, accounts for an increase of 0.65 in the maximum rate of change in glucose concentration under heat stress compared to optimal temperature, which is not significant at the 5% significance level (*p *= 0.2533). However, the β_2_.Heat parameter estimate is negative and larger than β_2_.Intercept, thereby implying that on average the time at which the rate curve attains half of its maximum due to heat stress is shorter than under optimal temperature conditions. The estimate is negative with a very low *p*-value (*p *< 10^-4^), which implies that glucose is taken up significantly faster during heat stress compared to the optimal temperature. The time interval remains the same for the optimal and the heat stress category, which indicates that the decay rate of the three parameter logistic curve is larger in the heat stress category. This conclusion is supported by the estimate of parameter β_3_.Heat, which is positive and significant in heat stress with a very small *p*-value. This result implies that the rate of decay during heat stress is faster than under optimal temperature conditions.

The effect of a return to the optimal temperature during recovery after heat stress is captured by the parameter estimates β_1_.Recovery β_2_.Recovery and β_3_.Recovery. The estimates are negative in sign with very low *p*-values implying that the glucose uptake profile during recovery has on average a lower maximum value as depicted by β_1_.Recovery, a shorter time period to attain half of its maximum (β_2_.Recovery) and a slower rate of decay (β_3_.Recovery). This translates into a flatter uptake rate curve during recovery as compared to the initial phase of optimal ambient temperature.

### Results of the Significance Analysis

Table 2 shows the results of an ANOVA for testing the effect of ambient temperature on the rate of change of glucose consumption over time. The likelihood ratio test results in a very small *p *-value (<0.0001), which again suggests with high confidence that temperature significantly affects the glucose uptake dynamics.

**Table 2:** Results of ANOVA and the overall likelihood ratio test for determining whether the effect of temperature on glucose uptake is significant.

Model	Df	AIC	BIC	logLik	Test	L.Ratio	*p*-value
Full	36	937.4367	1089.876	-432.7186			

Reduced	30	1117.0356	1244.068	-528.5178	1 vs 2	191.5988	<0.0001

For the second part of the analysis, testing the effect of heat preconditioning on the glucose uptake dynamics over time, the likelihood ratio test for the ANOVA results in a very small *p*-value (*p *= 0.0004) indicating that preconditioning significantly affects glucose uptake dynamics. This result also suggests that the three-parameter logistic mixed-effects model explains the glucose uptake characteristics for this situation as well.

To investigate the overall effect of preconditioning, we considered two models as before: a full model and a reduced model. In the full model the parameters obtained with nonlinear regression depend on preconditioning, and temperature, whereas in the reduced model the parameters depend only on temperature, which is accounted for as the primary covariate. The results are again significant (Table 3).

**Table 3:** Results of ANOVA and the overall likelihood ratio test for determining whether heat stress during growth significantly affects the glucose uptake dynamics.

Model	Df	AIC	BIC	logLik	Test	L.Ratio	*p*-value
Full	24	990.3122	1091.938	-471.1561			

Reduced	21	1002.7044	1091.627	-480.3522	1 vs. 2	18.3923	0.0004

We can furthermore investigate the interaction effect between temperature and preconditioning. In this case, the full model is formulated in such a fashion that the parameters of the model depend on temperature, preconditioning and the temperature-preconditioning interaction effect, whereas the parameters of the reduced model depend on temperature and preconditioning as the only fixed effects terms. Our results indicate that the interaction effect of temperature and preconditioning is highly significant (*p *< 0.0001) at the 5% significance level, which implies that preconditioning and later heat stress interact significantly and lead to a combined effect on the glucose uptake profiles (see Additional file 1 Table S5 in the Appendix for ANOVA results). The three-parameter logistic mixed-effects model again explains glucose uptake dynamics very well for this situation.

The effect of initial biomass on glucose uptake dynamics was investigated following a similar procedure as above. Our results show that the *p*-value for the likelihood ratio test is very small (p < 0.0001) indicating that the amount of cell biomass at harvest significantly affects glucose uptake kinetics (see Additional file 1 Table S6 in the Appendix for ANOVA results). For this scenario, the parameters of the full three-parameter logistic mixed-effects nonlinear regression model vary with respect to temperature and the amount of biomass harvested, while the parameters in the reduced model depend only on temperature.

### Pair-wise Comparisons

In addition to the overall assessments, it is also possible to execute pair-wise comparisons between different temperature conditions during glucose uptake. For these comparisons, one condition needs to be selected as reference, and as before we choose the optimal temperature for this purpose. The analysis is achieved with an approximate *F*-test for testing the joint significance of the terms in the models. The main results consist of very small *p*-values (*p *<0.0001) that indicate significant differences between all pairs of conditions (Table 4). The low *p*-values in all three cases demonstrate that the added temperature terms in the final models are highly significant in all cases. Interestingly, the glucose uptake dynamics also differs between optimal and recovery conditions.

**Table 4:** Approximate *F*-test; for joint significance of the fixed effects terms (temperature and preconditioning) in the model.

Fixed Effect	Comparison	NumDF	denDF	F-value	*P*-value
Temperature	Optimal & Heat Stress	3	325	118.6734	<0.0001

Temperature	Optimal & Recovery	3	331	51.3787	<0.0001

Temperature	Heat Stress & Recovery	3	331	56.1397	<0.0001

Preconditioning	Absence & Presence	3	499	4.6458	0.0033

The significance of including temperature in the model indicates that a substantial part of the type-to-type variation in the dynamics of glucose uptake is explained by pair-wise differences in temperature. In other words, the fixed effects terms introduced in the model to explain the temperature variability in the parameter estimates are significantly different from zero at the 5% significance level. Since the result of the *F*-test is highly significant, we can employ an additional *t*-test in order to identify exactly which parameters are significant and which are not in explaining the type-to-type variability in the model. Table 4 also identifies the *p*-value for the approximate *F*-test for testing the effect of preconditioning in the model as very small (p = 0.0033), which indicates that the fixed effect term (optimal or heat stress during growth), which was introduced into the model to explain type-to-type variability due to preconditioning, is significantly different from zero.

As indicated earlier, heat stress conditions during cell culture growth affect the dynamics of glucose consumption. In Table 5 the intercept terms (β_1_.Intercept, β_2_.Intercept, and β_3_.Intercept) represent the parameter estimates averaged over the six experiment types under optimal conditions, whereas the preconditioning terms (β_1_.Preconditioning, β_2_.Preconditioning, and β_3_.Preconditioning) represent the corresponding parameter estimates for comparing cell cultures grown under heat conditions relative to those grown under optimal conditions and averaged over the six experiment types. For instance, the parameter estimate β_1_.Preconditioning indicates the difference in the maximum value for the glucose uptake rate curve averaged over the six experimental types for cultures grown under heat conditions compared to cells grown under optimal temperature conditions. Its negative value indicates that the average maximum for the rate of glucose utilization is lower for preconditioned cells than for control cells grown under optimal condition, implying a lower uptake rate for cultures grown under heat conditions compared to those grown under optimal conditions. The parameter estimate β_2_.Preconditioning reflects the difference in the time at which the maximum rate of glucose utilization attains half of its value. Again, this value results from averaging over the experimental types for the preconditioned cells compared to controls. Its positive value indicates that, on average, the preconditioned cells take more time to consume glucose and hence had a slower glucose uptake than the control cells, which confirms a result from an earlier modeling study that used entirely different methods of analysis [4]. The parameter estimate β_3_.Preconditioning is the mean difference in the scale of glucose uptake for the cells grown under heat versus control conditions.

**Table 5:** Parameter estimates for the three-parameter logistic model with preconditioning included in the model as covariate.

Parameter	Estimate	t-value	*p*-value
β_1_.(Intercept)	11.5740	11.3163	0.0000

β_1_.Preconditioning	-3.0639	-1.7608	0.0789

β_2_.(Intercept)	-2.2904	-3.8724	0.0001

β_2_.Preconditioning	2.4782	2.3383	0.0198

β_3_.(Intercept)	-0.8490	-5.1658	0.0000

β_3_.Preconditioning	-0.6293	-1.9784	0.0484

Cell cultures grown under cold conditions are considered as the reference category.

### Results of the Simulation Study

In order to study the robustness of the parameter estimates using the nonlinear mixed-effects model for the rate of glucose uptake that was deemed best (the three-parameter logistic model) we used a simulation study. We checked the performance of the results over multiple variance-covariance matrices ψ and report the results for two representative cases where the variance-covariance parameter matrices are multiples of ψ, which were estimated directly from the data:

and

The estimated biases and standard deviations of the fixed effects parameters are reported in Tables 6 and 7 respectively. Estimated biases and standard deviations both remain quite small and are therefore acceptable. For other values of the parameters, the parameter estimates are also robust, which suggests that the method works reasonably well (results not reported).

**Table 6:** Parameter estimates for the simulation study with the three parameter logistic model with random effect generated from N (0, 0.001*estimated ψ from the data).

Parameter	Simulation parameter values	Simulated parameter estimate	Estimated Bias	Estimated Standard Error
β_1_.Intercept	10.4096	10.4321	-0.0225	0.0724

β_1_.Heat	0.6456	0.6146	0.0311	0.0956

β_1_.Recovery	-2.0912	-2.1051	0.0138	0.1058

β_2_.Intercept	-0.8622	-0.8665	0.0043	0.0347

β_2_.Heat	-0.9627	-0.9559	-0.0068	0.0399

β_2_.Recovery	-0.7040	-0.6986	-0.0055	0.0663

β_3_.Intercept	-1.0846	-1.1007	0.0161	0.0277

β_3_Heat	0.5000	0.5128	-0.0128	0.0320

β_3_.Recovery	-0.3239	-0.3332	0.0093	0.0524

**Table 7:** Parameter estimates for the simulation study with the three parameter logistic model with random effect generated from N (0, 0.01*estimated ψ from the data).

Parameter	Simulation parameter values	Simulated estimate	Estimated Bias	Estimated Standard Error
β_1_.Intercept	10.4096	10.3636	0.0460	0.0595

β_1_.Heat	0.6456	0.6663	-0.0207	0.0802

β_1_.Recovery	-2.0912	-2.0240	-0.0673	0.0853

β_2_.Intercept	-0.8622	-0.8440	-0.0182	0.0275

β_2_.Heat	-0.9627	-0.9732	0.0105	0.0320

β_2_.Recovery	-0.7040	-0.6898	-0.0143	0.0512

β_3_.Intercept	-1.0846	-1.1172	0.0326	0.0224

β_3_Heat	0.5000	0.5040	-0.0039	0.0246

β_3_.Recovery	-0.3239	-0.3288	0.0050	0.0387

## Discussion and Conclusions

We have developed stochastic non-linear regression models to fit and analyze sparse biological time course data. In the application shown here, these data were generated with NMR methods. They describe the dynamics of glucose uptake by yeast cells at different temperatures. Some of the cells in the study were grown under optimal control conditions, while others were preconditioned with heat treatment during growth. To facilitate strict comparisons, we used centered time, which took advantage of the fact that the time intervals for data collection were the same for all experimental set-ups and avoided problems caused by different starting times in the various experiments.

The main goal of the study was to develop rigorous statistical tests to assess the effects of temperature and preconditioning on the observed temporal glucose uptake profiles. To achieve this goal, we designed nonlinear mixed-effects regression models and analyzed them with customized ANOVA and maximum likelihood ratio tests.

The results indicate that a change in temperature from optimal to heat stress conditions (30°C to 39°C) produced significant differences in glucose uptake profiles. Specifically, the increase in temperature had a positive effect [14] on the rate of glucose uptake, while a corresponding decrease in temperature from heat stress to recovery resulted in a reduction in rate of glucose uptake. However, the recovery profiles were found to be different from the initial profiles (initial optimal condition), although the temperature was the same. We also determined that preconditioning with heat during cell growth resulted in significant differences in the glucose uptake profiles later in life. These differences presumably reflect a preparation of the cells to survive similar stresses [15,16] and [17] and to recover in a timely manner. From a methodological point of view, the results indicate that the methods described here are able to detect subtle difference in time course data from normal and stressed cells.

The statistical methods presented in this paper reach far beyond tests for the effects of different stress conditions. In fact, it appears that the exact same statistical procedures should be applicable and very useful for finding significant differences in any short biological time course data of a similar format. For instance, it seems that temporal expression profiles of genes or proteins from high-throughput experiments could be clustered by pathways and then analyzed with the proposed methods to discover differences between normal and disease conditions.

The estimation of parameter values from biological time series data is not a trivial problem, and the estimation algorithms for nonlinear mixed-effects are computationally complex and often provide less accurate inference than for linear effects models. Furthermore, nonlinear regression models require starting estimates for the fixed effects coefficients. Determining reasonable starting parameter estimates is somewhat of an art and not intuitive in many situations. Nevertheless, the concepts presented here are quite general, and algorithms exist in statistical software packages like R for their implementation and analysis. Finally, this type of analysis, like almost all dynamic modeling efforts in systems biology, requires the choice of a parametric model. It is unclear how one could get around this requirement, which is a challenge for about any modelling study in systems biology. One could potentially use canonical forms, such as piecewise power-law functions [18], but while these would be slightly more generic, they would still be parametric.

## Authors' contributions

JN performed the statistical analysis and contributed to the writing of the manuscript, especially with respect to the statistical methods and results. LLF executed all NMR experiments, under guidance from HS, and contributed to the interpretation of results. EOV conceived the study and oversaw the production of the manuscript. SD developed the statistical modeling and testing concepts, provided overall guidance, and contributed to the writing of the manuscript. All authors read and approved the final manuscript.

## Supplementary Material

Additional file 1 **Appendix**. This file provides additional model comparisons tables. These tables explain the choice of best model, the test of interaction effect, the test of biomass concentration as well as comparing the two and three parameter exponential models. Click here for file
