# Supplementary material for: Statistical Inference Methods for Sparse Biological Time Series Data
Source: BMC Syst Biol. 2011 Apr 25;5:57. doi: 10.1186/1752-0509-5-57 (PMC3114728; doi:10.1186/1752-0509-5-57)
Supplement: Additional file 1 — Appendix. This file provides additional model comparisons tables. These tables explain the choice of best model, the test of interaction effect, the test of biomass concentration as well as comparing the two and three parameter exponential models. [file 1752-0509-5-57-S1.DOC]

Appendix: Additional Model Comparisons

1. In order to test whether a three-parameter exponential decay model (model 2) would outperform a regular two-parameter exponential model (model 1), we analyzed both models with respect to all data. The results (Table S1) indicate that the more complex model is preferable.

**Table S1**: Comparison of two- and three-parameter exponential decay models

| **Model** | **Df** | **AIC** | **BIC** | **logLik** | **Test** | **L.Ratio** | ***p*-value** | **Residual Std dev** | **Data count** |
| --- | --- | --- | --- | --- | --- | --- | --- | --- | --- |
| Model 1 | 6 | 2410.594 | 2436.001 | -1199.297 |  |  |  | 2.4876 | 510 |
| Model 2 | 7 | 2330.412 | 2360.053 | -1158.206 | 1vs2 | 82.1824 | <.0001 | 2.2737 | 510 |

2. We compared exponential and three- and four-parameter logistic models (Tables S2, S3, and S4). These comparisons showed that there is a significant difference between the three-parameter logistic model and the three-parameter exponential decay model (*p*-value of the likelihood ratio test in Table S2 below is <0.0001). We also compared the three-parameter exponential decay model with the four-parameter logistic model and we found significant differences between the two models as evidenced by the low p-value (<0.0001). Moreover, we checked the differences between the three- and four-parameter logistic models: these do not exhibit significant statistical difference between them. However, after checking the AIC and BIC values of all models we selected the three-parameter logistic model (AIC and BIC are both smallest).

**Table S2**: Comparison of the three-parameter logistic model with the exponential uptake model

| **Model** | **df** | **AIC** | **BIC** | **logLik** | **Test** | **L.Ratio** | ***p*-value** |
| --- | --- | --- | --- | --- | --- | --- | --- |
| Three-parameter logistic | 10 | 1911.637 | 1953.981 | -945.8184 |  |  |  |
| Exponential uptake model | 7 | 2330.412 | 2360.053 | -1158.2059 | 1 vs 2 | 424.775 | <0.0001 |

**Table S3: Comparison of the four-parameter logistic model with the exponential uptake model**

| **Model** | **df** | **AIC** | **BIC** | **logLik** | **Test** | **L.Ratio** | ***p*-value** |
| --- | --- | --- | --- | --- | --- | --- | --- |
| Four parameter logistic | 15 | 1918.265 | 1981.781 | -944.1323 |  |  |  |
| Exponential uptake model | 7 | 2330.412 | 2360.053 | -1158.2059 | 1vs2 | 428.1471 | <0.0001 |

**Table S4: Comparison of the three-parameter logistic model with the four-parameter logistic model**

| **Model** | **df** | **AIC** | **BIC** | **logLik** | **Test** | **L.Ratio** | ***p*-value** |
| --- | --- | --- | --- | --- | --- | --- | --- |
| Three parameter logistic | 10 | 1911.637 | 1953.981 | -945.8184 |  |  |  |
| Four parameter logistic | 15 | 1918.265 | 1981.781 | -944.1323 | 1vs2 | 3.372077 | 0.6428 |

3. We present the ANOVA results (Tables S5 and S6) for testing the interaction effect as well as the effect of initial biomass on glucose uptake dynamics.

**Table S5: Results of ANOVA and the overall likelihood ratio test for determining whether** the temperature-preconditioning interaction effect significantly affects glucose uptake dynamics

| **Model** | **Df** | **AIC** | **BIC** | **logLik** | **Test** | **L.Ratio** | ***p*-value** |
| --- | --- | --- | --- | --- | --- | --- | --- |
| Full | 30 | 886.8872 | 1013.919 | -413.4436 |  |  |  |
| Reduced | 24 | 990.3122 | 1091.938 | -471.1561 | 1 vs 2 | 115.4250 | <0.0001 |

**Table S6: Results of ANOVA and the overall likelihood ratio test for determining whether** the effect of the amount of initial biomass significantly affects glucose uptake dynamics

| **Model** | **Df** | **AIC** | **BIC** | **logLik** | **Test** | **L.Ratio** | ***p*-value** |
| --- | --- | --- | --- | --- | --- | --- | --- |
| Full | 36 | 937.4367 | 1089.876 | -432.7184 |  |  |  |
| Reduced | 21 | 1002.7044 | 1091.627 | -480.3522 | 1 vs 2 | 95.2677 | <0.0001 |
